# Editorial: Fighting for recovery on multiple fronts in spinal cord injury

**DOI:** 10.3389/fncel.2023.1178192

**Published:** 2023-03-14

**Authors:** Jacob Kjell, Jennifer N. Dulin, Philippa M. Warren

**Affiliations:** ^1^Department of Clinical Neuroscience, Karolinska Institutet (KI), Stockholm, Sweden; ^2^Texas A&M Institute for Neuroscience, Texas A&M University, College Station, TX, United States; ^3^Department of Biology, College of Science, Texas A&M University College Station, College Station, TX, United States; ^4^Wolfson Centre for Age-Related Diseases, School of Neuroscience, King's College London, London, United Kingdom

**Keywords:** spinal cord injury, functional recovery, pathobiology, therapeutic treatment, clinical trial

Currently, no approved therapies exist to restore movement, sensation, or autonomic function to the 27 million people world-wide who suffer from a spinal cord injury (SCI). To rectify this, we need to better understand the vast array of processes and pathophysiologies that contribute to this multifaceted disorder. There is significant reason for optimism: the past decade has witnessed a vast increase in knowledge fostered by innovations in research technologies which enable us to understand the nature of the disorder and its underlying mechanisms. Slowly but surely, the promise of this research is being realized through the development and clinical assessment of SCI treatments and assistive technologies to aid the quality of patients' lives. Within this Research Topic, “*Fighting for recovery on multiple fronts in spinal cord injury*,” we have brought together basic and clinical studies to review the current state of the field, discuss the outcomes of clinical trials, and highlight innovative research which together will assist in deciphering the mechanisms of dysfunction, recovery, and treatment after SCI.

The wealth of evidence that treatment effect may be altered due to sex differences has led national funding bodies in the United Kingdom and beyond to require pre-clinical research be conducted on mixed sex populations. Stewart et al. have produced a compelling review suggesting that age may similarly affect an individual's response to SCI treatment. The authors provide a considered and powerful argument utilizing both clinical and basic research, advocating for the use of age as a variable in pre-clinical models. They conclude age may affect SCI treatment outcome and injury pathophysiology, and may need to be accounted for when translating strategies into the clinic. Similarly important for therapeutic potential, Wulf and Tom provide an extensive review of the effect SCI has upon the sympathetic nervous system, concentrating on the impact upon critical organ function. The authors provide persuasive evidence detailing the importance of restoring sympathetic nervous system function to aiding SCI patient health and quality of life. The critical and wide-ranging importance of astrocyte heterogeneity to SCI is appraised by Yu et al. demonstrating potential flaws in current classification systems. They discuss the multiple roles of astrocytes following injury as regulators of inflammation and a component in astrogliosis and subsequently how these glial cells may be targeted as a treatment for SCI recovery. The extracellular matrix is another important element with multiple roles in both the injured and uninjured spinal cord. Sánchez-Ventura et al. presented a review on the perineuronal nets (PNN), focusing on the potential for targeted modulation and its consequences. There authors highlight several crucial knowledge gaps and discuss insights that can be drawn from development and plasticity in order to find more effective PNN-targeting strategies.

There is an increasing number of clinical trials for SCI providing evidence concerning what impacts patient recovery while also continuously amassing experiences that may guide researchers and clinicians in future practice and treatment translation. Kjell and Svensson share a perspective on the clinical advantages and opportunities of autologous peripheral nerve graft (PNG) therapy for SCI and put PNG therapy in context of our current understanding of SCI recovery and repair. They authors highlight the unique potential of PNGs to repair long tracts and be optimally surgically positioned within the spinal cord, an advantage that may also be exploited in combination with other therapeutic strategies. In clinical trials, primary outcome can be affected and obscured by secondary complications. Franz et al. perform a longitudinal study providing data describing the effect on clinical recovery of the secondary complication heterotopic ossification, common in SCI patients. The heterotopic ossification primarily occurred at the hip joints within 3 months after injury and the authors emphasize that early prevention or treatment needs to be established in order to safeguard functional recovery in patients. Finally, Dietz et al. performed a systematic review of 1,149 SCI clinical trials, and report how trial interventions and outcomes have evolved over time. Among other trends, the authors show that as the numbers of new clinical trials grow each year, studies focused on neuromodulation and rehabilitation dominate the clinical trial landscape. They also identify problems with low reporting of clinical trial results, highlighting a need for better reporting standards for SCI clinical trials.

This Research Topic also includes new original research utilizing experimental SCI models. First, Romanelli et al. utilized human mesenchymal stromal cell-derived extracellular vesicles (EVs) as an anti-inflammatory therapeutic intervention for SCI in rats. The authors found that intralesional, but not systemic, injection of EVs into sites of thoracic contusion improved long-term hindlimb motor functional outcomes and reduced thermal hypersensitivity. Markers of molecular and cellular inflammation were found to be reduced in the EV-treated injured spinal cord tissue, and lesion volume as well as axon tract integrity were improved vs. vehicle-treated animals. Together, these findings suggest a neuroprotective, anti-inflammatory effect of EV treatment. Further, a study by Kondo et al. examines the effects of treadmill training on locomotor recovery in a marmoset SCI model. For this study, the authors developed a new locomotor scoring system, the Marmoset Motor Scale for Locomotion. The authors show that treadmill training improves locomotor recovery in marmosets, with improved frequency of stepping, coordination, and kinematic trajectories vs. untrained animals. Training also induced changes in hindlimb representation within cortical motor maps, with greater cortical area capable of eliciting hindlimb movement. Collectively, these new studies shed new light on mechanisms of two different promising therapeutic interventions for SCI.

In this Research Topic, we highlight that SCI presents great challenges on both microscopic and macroscopic levels and is indeed a challenge on multiple fronts. The contributing authors have highlighted several of the challenges and aspects related to making the trip from “bench to bedside.” These contributions exemplify and make it salient that it is only by understanding these challenges that we may find future therapies that are able to improve functional recovery following SCI.

## Author contributions

All authors listed have made a substantial, direct, and intellectual contribution to the work and approved it for publication.

